# Leveraging Patient Preference Information in Medical Device Clinical Trial Design

**DOI:** 10.1007/s43441-022-00450-9

**Published:** 2022-08-27

**Authors:** Liliana Rincon-Gonzalez, Wendy K. D. Selig, Brett Hauber, Shelby D. Reed, Michelle E. Tarver, Shomesh E. Chaudhuri, Andrew W. Lo, Dean Bruhn-Ding, Barry Liden

**Affiliations:** 1 Medical Device Innovation Consortium, 1655 N Ft. Myer Drive, 12th Floor, Arlington, VA 22209 USA; 2 WSCollaborative, McLean, VA USA; 3 Pfizer, New York, NY USA; 4 CHOICE Institute, University of Washington School of Pharmacy, Seattle, WA USA; 5 Duke Clinical Research Institute, Duke University, Durham, NC USA; 6 Food and Drug Administration, Center for Devices and Radiological Health, Silver Spring, MD USA; 7 Quantitative Life Sciences Advisors, Cambridge, MA USA; 8 Laboratory for Financial Engineering Department of Electrical, Engineering and Computer Science Sloan School of Management; and Computer Science and Artificial Intelligence Laboratory, MIT, Cambridge, MA USA; 9 Santa Fe Institute, Santa Fe, NM USA; 10 CVRx, Inc, Minneapolis, MN USA; 11 USC Schaeffer Center for Health Policy & Economics, Los Angeles, CA USA

**Keywords:** Bayesian decision analysis, Medical device, Clinical trials, Patient centricity, Stated-preference research, Regulatory policy

## Abstract

Use of robust, quantitative tools to measure patient perspectives within product development and regulatory review processes offers the opportunity for medical device researchers, regulators, and other stakeholders to evaluate what matters most to patients and support the development of products that can best meet patient needs. The medical device innovation consortium (MDIC) undertook a series of projects, including multiple case studies and expert consultations, to identify approaches for utilizing patient preference information (PPI) to inform clinical trial design in the US regulatory context. Based on these activities, this paper offers a cogent review of considerations and opportunities for researchers seeking to leverage PPI within their clinical trial development programs and highlights future directions to enhance this field. This paper also discusses various approaches for maximizing stakeholder engagement in the process of incorporating PPI into the study design, including identifying novel endpoints and statistical considerations, crosswalking between attributes and endpoints, and applying findings to the population under study. These strategies can help researchers ensure that clinical trials are designed to generate evidence that is useful to decision makers and captures what matters most to patients.

## Introduction

A rigorous clinical trial process is often a critical component for developing the evidence needed to ensure that a medical device is safe and effective in US regulatory reviews [1]. However, some have suggested that traditional endpoints used to evaluate new technologies may not always reflect the priorities of the patient [2]. One way to understand those priorities is to collect patient preference information (PPI), defined by the US Food and Drug Administration (FDA) as “qualitative or quantitative assessments of the relative desirability or acceptability to patients of specified alternatives or choices among outcomes or other attributes that differ among alternative health interventions” [3]. Development of scientifically robust quantitative assessments of patient preferences offers the opportunity for researchers and regulators to incorporate what matters most to patients into the product development and evaluation process [4].

Incorporation of PPI into clinical trial design is an emerging and evolving endeavor. A key benefit of PPI is that it can help inform the ongoing debate about the relative weights given to different outcomes in product evaluation. PPI allows clinical trialists to evaluate the importance of a wide array of outcomes from the patient perspective. Increasingly, stakeholders are beginning to understand that patient preferences and clinical outcome assessments are complementary, and PPI provides an opportunity to use scientifically sound methods to improve alignment among clinicians, regulators, and the specific patient population under consideration [5].

Leaders in the medical device field have developed a series of resources aimed at supporting efforts to collect and incorporate PPI throughout the lifecycle of a medical product. In 2015, the medical device innovation consortium (MDIC), a public–private partnership focused on advancing medical device regulatory science for patient benefit, published a seminal report on incorporating PPI into benefit-risk assessments with its patient-centered benefit-risk (PCBR) Framework [6]. While the PCBR Framework focused specifically on informing the assessment of benefit-risk tradeoffs within medical device reviews, the authors also envisioned the opportunity for industry sponsors to use PPI in the design of clinical trials [7].

Since 2015, MDIC has brought together organizations such as FDA, industry, and patients to advance a series of projects aimed at the development of case examples illustrating how PPI could be used to inform clinical trial design [6, 8–11]. This effort resulted in the development of the “Patient preference information in the design of clinical trials (PPI-CT) Framework” [12]. This paper summarizes the lessons learned during the development of the Framework, including a discussion of practical applications of Bayesian decision analysis (BDA) with quantitative patient preference data to facilitate the development of more patient-centric statistical designs for clinical trials.

## Methods

MDIC sought to develop additional resources for medical device researchers to understand the benefits of and approaches to using PPI in clinical trial design. MDIC convened a working group of 17 representatives of medical device companies, regulators, and patient advocates. Five case studies (Table 1) were selected from a convenience sample of PPI studies performed by working group members, including the two studies carried out by MDIC multistakeholder working groups, one by FDA, and two industry sponsored studies.

**Table 1:** Five Case Examples of Using PPI in Clinical Trial Design (compiled and assessed by MDIC [12])

Title	Researcher	Focus
Patient-centered outcomes research project for parkinson’s disease	MDIC in collaboration with FDA-CDRH, the Michael J. fox foundation for Parkinson’s research (MJFF), Massachusetts institute of technology (MIT), and RTI health solutions	A patient preference study was conducted in PD and the PPI was incorporated in a BDA tool as an explicit means to set significance levels during clinical trial design [13, 14]
Quantifying benefit-risk preferences for heart failure devices: a stated-preference study	MDIC & six device sponsor companies in collaboration with FDA-CDRH^a^, Duke clinical research institute, and QLS advisors consulting group	A patient preference study examined the acceptability of risks and benefits associated with devices used for HF, rather than for a specific device development program [15] A follow-on project computed the BDA-optimal statistical significance threshold that maximizes the expected value to patients with HF [16]
Selecting endpoints for clinical trials (heart failure)	Abbott Vascular	This project provided quantitative PPI on benefit-risk tradeoffs relevant to transcatheter mitral valve repair versus medical therapy for patients with HF and symptomatic secondary mitral regurgitation [17]
Establishing clinically relevant trial/study thresholds for success using PPI data	Edwards Lifesciences	PPI and a BDA model were planned to identify the optimal non-inferiority margin and statistical significance threshold from a patient perspective for an ongoing clinical trial [18, 19]
A PPI-based model for determining endpoint thresholds	FDA-CDRH	A proof-of-principle study was conducted on the preferences of obese participants regarding the use of medical devices to achieve weight loss and the results were retrospectively analyzed using a BDA model [20, 21]

*BDA* Bayesian design analysis, *FDA-CDRH* food and drug administration-center for devices and radiological health, *HF* heart failure, *MDIC* medical device innovation consortium, *PD* parkinson’s disease, *PPI* patient preference information

^a^Funded partially through a broad agency announcement grant from FDA-CDRH

The working group convened clinical investigators, industry trial designers, regulatory officials, patient preference experts, statisticians, and case studies leads to review the cases and identify key considerations when leveraging PPI in clinical trial design. These experts were individually interviewed by two of the authors to capture the lessons learned and to gather input and guidance about the most relevant topics and considerations. The 60 min interviews were tailored to the interviewee level of expertise and involvement in the case studies (if any). The working group then reviewed the findings to develop the PPI-CT Framework. The content of the PPI-CT Framework was also circulated among these experts and additional experts for review.

The resulting MDIC PPI-CT Framework [12] includes detailed discussions about planning timelines, budgeting expenses, engaging appropriate expertise, ensuring that PPI is collected from a representative sample of patients, and working with patient advocacy organizations.

The top five key topics relevant to the use of PPI in clinical trials designed for medical product development were chosen by the working group for further discussion in this paper, including the selection of novel endpoints, alignment between patient preference study attributes and traditional clinical trial endpoints, statistical considerations, and appropriateness of surveyed populations.

## Results

The experts identified several key considerations for industry sponsors and regulators interested in applying PPI to clinical trial design, including: (1) pursuing existing opportunities to work with regulators to incorporate PPI in regulatory decision-making, (2) identifying novel endpoints for patient preference studies, (3) aligning on the “crosswalk” between attributes selected for a patient preference study and endpoints used in a clinical trial, (4) ensuring the applicability of PPI to the specific population who will use the medical device under study, and (5) applying the most appropriate methods to leverage PPI to inform the statistical evaluation of trial data.

### Gauging Regulatory Interest

The US regulatory community has expressed interest in the inclusion of PPI in regulatory decision-making. Specifically, the US FDA Center for Devices and Radiological Health (CDRH) strongly supports engaging patients, collecting their perspectives, and advancing appropriate use of PPI within the device development and regulatory review processes. CDRH’s review staff, with the support of staff with expertise in patient preference study design and interpretation, regularly work with researchers to give feedback and guidance on the conduct of patient preference studies to collect PPI for use in regulatory evaluation [22–24]. CDRH has issued guidance documents to support integration of PPI in medical device development and evaluation and has actively indicated a willingness to engage directly with researchers on these initiatives [25–33].

A key ingredient for success in these efforts is early buy-in from critical stakeholders about the importance of ensuring that a clinical trial design reflects the tradeoffs patients are willing to accept as a function of the magnitude of gains and decrements in health. Involving these stakeholders helps to ensure that patient preference studies are designed and positioned to collect PPI that best addresses the pertinent research questions.

### Identifying Novel Endpoints

A critical element of designing a patient preference study that will inform the design of a clinical trial is to select the most appropriate attributes (i.e., outcomes, characteristics, or qualities that are inherent to a disease or treatment options being evaluated) that relate to safety and effectiveness outcomes or endpoints in the clinical trial. Endpoints are most useful as attributes in a patient preference study when they can be translated into attributes that are understandable and meaningful to patients [34]. Once attributes that matter to patients (including potentially different sub-groups of patients) have been identified through patient preference studies or other approaches, researchers can work with their clinical trial teams and regulators to appropriately incorporate them as endpoints in the clinical trial.

If a patient preference study is executed early enough in the product development process, it presents a key opportunity for leveraging the PPI collected to inform clinical trial endpoint identification and prioritization. At early stages of development, a “bottom-up” approach should be used to identify and develop endpoints that would be meaningful to patients, without being limited by existing endpoints for a certain device category. In one case example, patient advisors/patient scientists involved in a Parkinson’s disease patient preference study identified psychological, cognitive, and pain outcomes as important treatment considerations [13]. While Parkinson’s-related pain was reported in the literature, it was rarely emphasized relative to other disease symptoms; accordingly, the clinical research community does not routinely measure Parkinson’s-related pain as a trial endpoint. The patient preference study findings provided evidence to support the inclusion of pain as a patient-relevant endpoint in a subsequent device development clinical trial. PPI could also help inform medical device development for the subset of patients with Parkinson’s disease for whom the treatment of motor symptoms alone results in residual unmet patient need.

### Aligning Patient Preference Study Attributes and Traditional Clinical Trial Endpoints

An attribute that is determined to be a high priority for patients relevant to the clinical context (e.g., survival, pain) and impacted by the device under study could be incorporated as a primary or secondary clinical trial endpoint, where appropriate. However, in some cases, existing clinical trial endpoints do not map to a patient-prioritized attribute, or the construct represented by the attribute may be difficult to measure (e.g., independence). In addition, it can be challenging to develop a clear “crosswalk”—where a direct connection can be made between an attribute within a patient preference study and a traditional clinical trial endpoint—in cases where the trial endpoint has already been deemed essential to device evaluation or is a composite of multiple endpoints. Identification of specific elements of a composite endpoint to define attributes for the patient preference study could be discussed with regulators, with the goal of aligning on an approach to evaluate and weight measurable factors (often symptoms) that matter most to patients [35–38]. It is important to note that patient preference studies typically do not define the entire sphere of relevant endpoints; rather, they may suggest additional outcomes or help to prioritize outcomes already identified as relevant.

### Ensuring Applicability of PPI to the Population Under Study

To ensure that PPI is reflective of the intended population for a potential medical device, it is important that the participants in the patient preference study accurately reflect the population that would be indicated to use the medical device under development [34]. For this reason, recruiting participants for a patient preference study in a timely and cost-effective way requires accurate reporting by and about the patients from whom PPI is to be generated. However, not all patients can accurately report on details about their condition [39, 40] as disease sub-types, specific aspects, or stages of the condition can make this undertaking complicated. For this reason, one approach used in the case studies was to engage patients who have been referred by a physician or whose electronic medical record included the specific diagnosis that the device under development is intended to treat. This is often referred to as a “confirmed diagnosis” [41].

Because obtaining confirmed diagnoses can be time consuming and costly, patient preference studies may also collect data from individuals who “self-report” or self-identify that they have a certain condition. In these cases, researchers may want to look for secondary data (e.g., the channel through which a patient was contacted, such as a patient organization network) and supporting information from participants (e.g., information about their symptoms or treatments that may be unique to the relevant condition) that can be used to increase confidence about the diagnosis [42]. Depending on the condition under study, self-report may be a justifiable approach to identifying participants for a patient preference study [43, 44].

### Applying Appropriate Statistical Methods

It may be possible to leverage PPI to inform statistical considerations during clinical trial design (e.g., sample size, significance threshold, power) and allow the execution of a more targeted clinical study that is focused on outcomes and levels of certainty, resulting in a more efficient trial from the patient perspective [45, 46].

Patients with a serious medical condition, rapid disease progression, and/or lack of effective therapies may be willing to accept more uncertainty about the benefits and risks of using a new device in exchange for having access to it sooner. In this case, it may be preferable, from the patient perspective, to design a clinical trial with a smaller sample size that would allow the study to be completed in a shorter timeframe. This trial design may also incorporate a higher level of statistical uncertainty [46]. On the other hand, patients with a less severe condition, chronic illness, and access to existing treatments may prefer an approach with less uncertainty, even if it delays access to the new device. To address these scenarios, researchers at MIT developed a statistical framework to incorporate patient preferences into trial design [46]. The Bayesian decision analysis (BDA) framework provides a systematic, quantitative, patient-centered, and transparent approach to setting the statistical significance threshold for a clinical trial. The methodology attempts to balance the consequences of approving an ineffective and possibly harmful treatment (false approval) against the consequences of rejecting an effective treatment (false rejection) such that the overall expected utility of a clinical trial is maximized. For example, if we set the significance level to be more stringent, we reduce the chance of a false approval, but increase the chance of a false rejection. Moreover, large clinical trials may provide more evidence, yet may unintentionally delay patient access to effective treatment because larger trials typically take longer to complete [45–48]. In this way, the overall consequences of approving an ineffective and possibly harmful treatment are balanced against the consequences of rejecting a safe and effective treatment using insights gathered from the patient population under study [47]. In addition to incorporating PPI related to risk-tolerance among patients, the BDA framework can also analyze tradeoffs relating to time preferences among patients (e.g., how long would patients be willing to wait for a novel device). This framework might be particularly valuable in diseases for which clinical trial recruitment can be challenging, such as rare disease or conditions with a high mortality rate.

## Discussion

The case studies reviewed in the PPI-CT Framework [12], when taken together, provide researchers with some valuable insights on how to develop PPI that may be useful for informing clinical trial design. For example, leveraging patient input from patient advisors and patient scientists involved in the Parkinson’s study led to inclusion of Parkinson’s-related pain as an attribute, which is rarely used as a trial endpoint. Another case study recruited both a web panel (self-reported diagnosis) and a confirmed diagnosis sample for a patient preference study in heart failure. The results of that study showed that both samples had similar benefit-risk preferences.

Uptake of the BDA framework approach by manufacturers and trialists for weighing tradeoffs (by applying the perspectives of patients, clinicians, and regulators in calculating the optimal significance level) has been limited. One barrier is the lack of existing, high-quality, relevant PPI data. There is a continuing need to conduct and report PPI to develop a future landscape of existing high-quality data that can be used to support these approaches. In the device arena, more work is needed to further expand these efforts, including projects that tie directly to a device clinical development program and subsequent regulatory review.

It may be beneficial to include the acquisition of PPI within a clinical trial plan or in a parallel protocol using an external sample and have the analysis plan prospectively incorporated into the clinical trial statistical analysis plan (SAP) to support clinical assumptions used to design the trial. Additional evaluation is needed to identify the best approaches to use when PPI is not available to a researcher in the early development stages. One way to incorporate PPI in later stages is to take advantage of an adaptive clinical trial design, where PPI is used as input for a prospectively planned trial modification [49]. Such a protocol would allow the trial to end early in cases where the investigational treatment shows clear signs of effectiveness or ineffectiveness for patient-relevant endpoints, or to continue enrollment and collection of follow-up data to allow more definitive conclusions to be made about a device.

Additional research is needed to create a crosswalk between patient-reported outcomes used as clinical trial endpoints and PPI attributes used in patient preference studies [50, 51].

A key consideration for clinical development teams is understanding the tradeoff between costs and benefits for collecting and implementing PPI as part of their trial design efforts. Additional evidence regarding the return on investment will be needed to demonstrate the value of this approach overall.

Other topics that may benefit from additional work include the expansion of techniques to enhance diversity of patient perspectives in patient preference studies to ensure representativeness. There is also an opportunity to engage in discussions with payors about incorporating PPI in their decision-making about coverage and reimbursement, as these can be key hurdles to patient access and could preclude patients from receiving a much-needed treatment.

## Conclusion

While the use of PPI in the design of clinical trials has been proposed and advocated for more than a decade, specific examples and practical applications have not been available until now. This paper and the corresponding PPI-CT Framework [12] provide clinical trial researchers and teams with the opportunity to incorporate lessons learned from case studies into their development programs. As the body of evidence supporting the feasibility and value of patient preference studies and PPI grows, stakeholders will develop additional confidence and comfort with using PPI to design the most streamlined and efficient clinical trials possible, yielding outcomes that systematically include the benefit-risk preferences of patients. The authors hope the practical considerations discussed within this paper and the PPI-CT Framework [12] will support advances in this area.
